# Supplementary material for: Ultra-sensitive molecular residual disease detection through whole genome sequencing with single-read error correction
Source: EMBO Mol Med. 2024 Aug 20;16(9):2188–209. doi: 10.1038/s44321-024-00115-0 (PMC11393307; doi:10.1038/s44321-024-00115-0)
Supplement: Supplementary file 9 — Appendix [file 44321_2024_115_MOESM9_ESM.pdf]

Ultra-sensitive molecular residual disease detection through whole genome sequencing with single-read error correction

Xinxing Li[1†], Tao Liu[2†], Antonella Bacchiocchi[3†], Mengxing Li[4†], Wen Cheng[4], Tobias Wittkop[5], Fernando L. Mendez[5], Yingyu Wang[5], Paul Tang[5], Qianqian Yao[6], Marcus W. Bosenberg[3,7,8], Mario Sznol[7,9], Qin Yan[7,8,10], Malek Faham[5], Li Weng[5*], Ruth Halaban[3,7*], Hai Jin[4*], Zhiqian Hu[1,11*]

Table of Content **Page**

Figure	Page
**Appendix Figure S1. The error rates of 200Mb randomly selected positions in three healthy individuals through 20 iterations.**	**1**
**Appendix Figure S2. The representative profile of variant types for colorectal cancer patients.**	**1**
**Appendix Figure S3. Observed circulating variant allele frequency point estimates in simulated subsamples of data from serial dilutions of three healthy cfDNA.**	**2**
**Appendix Figure S4. Number of observed variant molecules in serial diluted melanoma cfDNA samples and healthy controls.**	**3**
**Appendix Figure S5. The observed specificity shown as boxplots for each equivalent variant count number (2K, 5K, 10K, and 20K) in 117 mismatched plasma samples from 57 patients.**	**4**
**Appendix Figure S6. Distribution of probability and expected number of observed variants in Poisson model at different allele frequencies.**	**5**

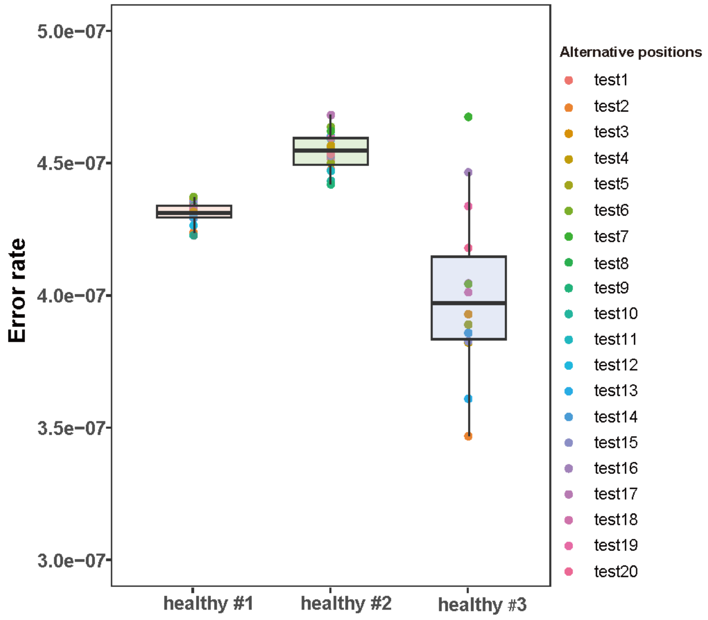

Appendix Figure S1. The error rates of 200Mb randomly selected positions in three healthy individuals through 20 iterations.

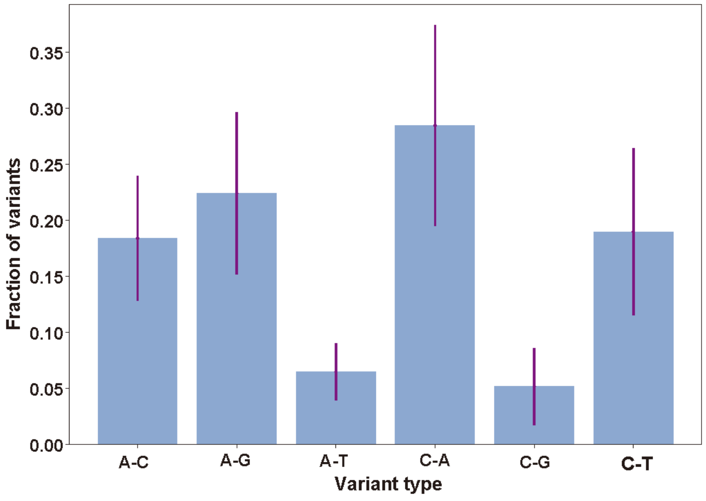

Appendix Figure S2. The representative profile of variant types for colorectal cancer patients. Point estimates are obtained as the fraction of each variant type in the aggregation of variants from multiple colorectal cancer patients. Error bars are one standard deviation using the distribution of fractions in all patients.

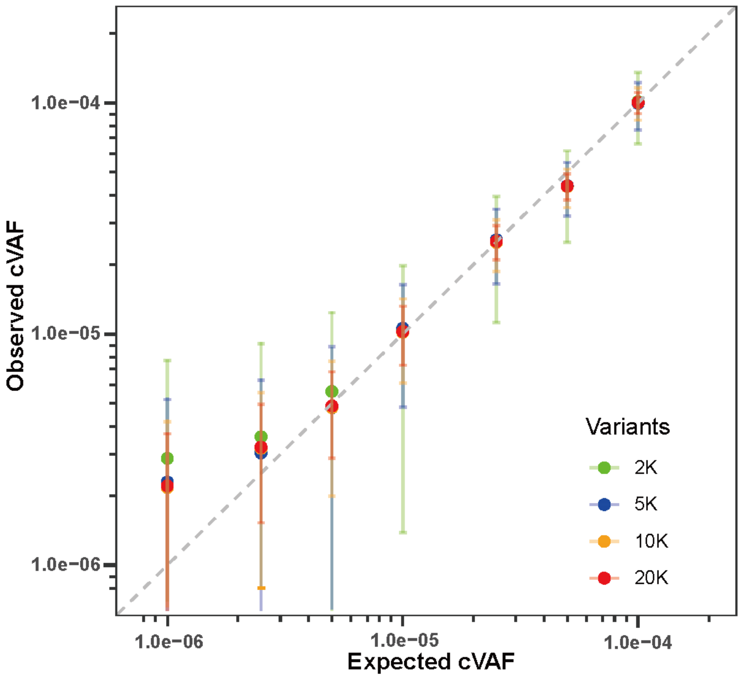

Appendix Figure S3. **Observed circulating variant allele frequency point estimates in simulated subsamples of data from serial dilutions of three healthy cfDNA.** It was performed in 1000 simulations per concentration and number of variants. Expected circulating variant allele frequency (cVAF) ranged from 1×10^{-4} to 1×10^{-6}. Dots represent the mean of the point estimate, and the bars indicate 1 standard deviation.

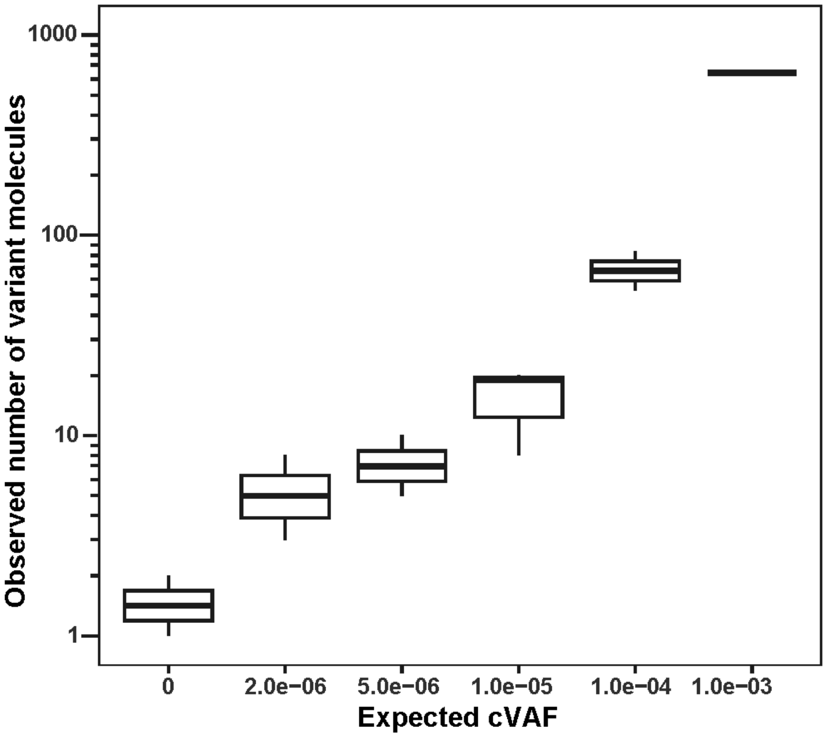

Appendix Figure S4. Number of observed variant molecules in serial diluted melanoma cfDNA samples and healthy controls. Experiments were performed with one test at 1×10^{-3}, two replicates at 1×10^{-4}, three replicates at 1×10^{-5}, 5×10^{-6}, 2×10^{-6}, and two replicates with the healthy control sample.

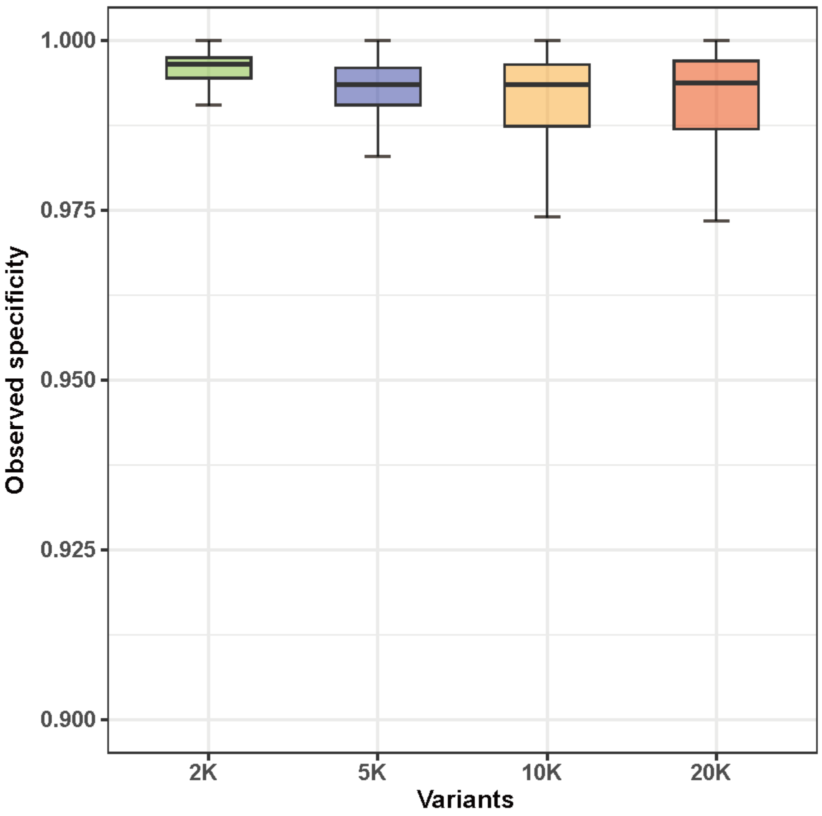

Appendix Figure S5. The observed specificity shown as boxplots for each equivalent variant count number (2K, 5K, 10K, and 20K) in 117 mismatched plasma samples from 57 patients. The average specificity across all plasma samples is above the nominal specificity of 99% set in the model.

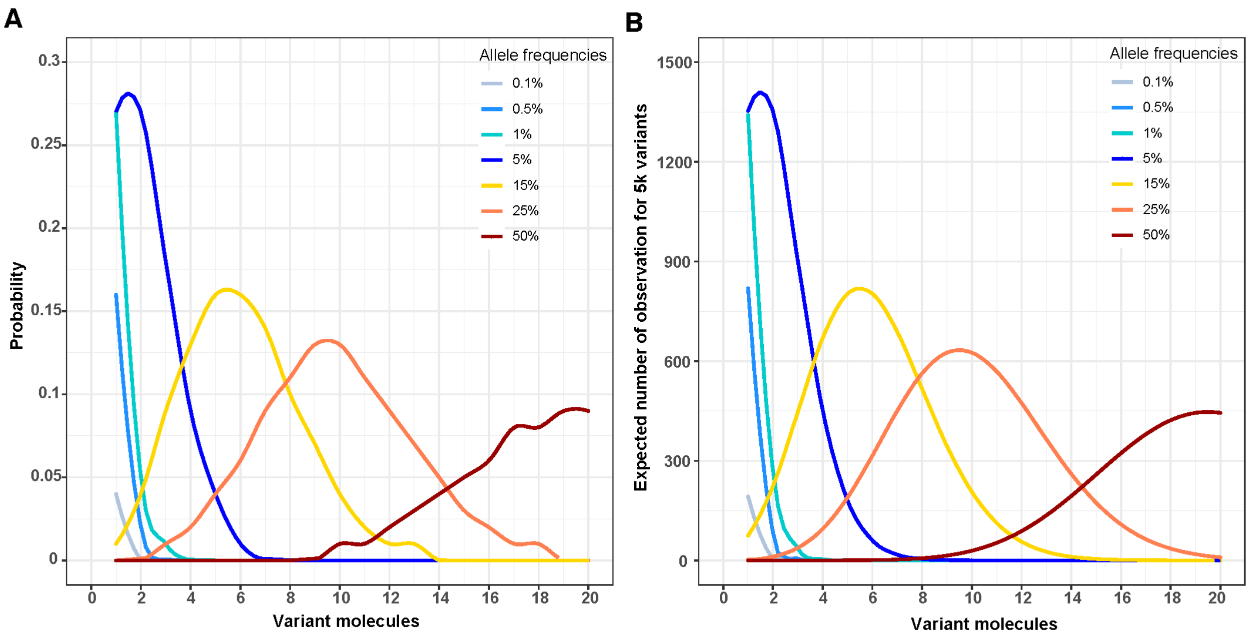

Appendix Figure S6. Distribution of probability and expected number of observed variants in Poisson model at different allele frequencies.

A Probability distribution of Poisson model using depth of 40 and circulating variant allele frequencies (cVAF) ranging from 0.1% to 50%. X-axis is the number of observed variant molecules and y-axis is the probability to see this many variant molecules for a given variant for different cVAFs.

B Similar to A but showing the expected number of observed variants under the assumption of evaluating 5K variants as tumor-specific markers.
