# Supplementary material for: Ultra-sensitive molecular residual disease detection through whole genome sequencing with single-read error correction
Source: EMBO Mol Med. 2024 Aug 20;16(9):2188–209. doi: 10.1038/s44321-024-00115-0 (PMC11393307; doi:10.1038/s44321-024-00115-0)
Supplement: Supplementary file 15 — Expanded View Figures [file 44321_2024_115_MOESM15_ESM.pdf]

Expanded View Figures

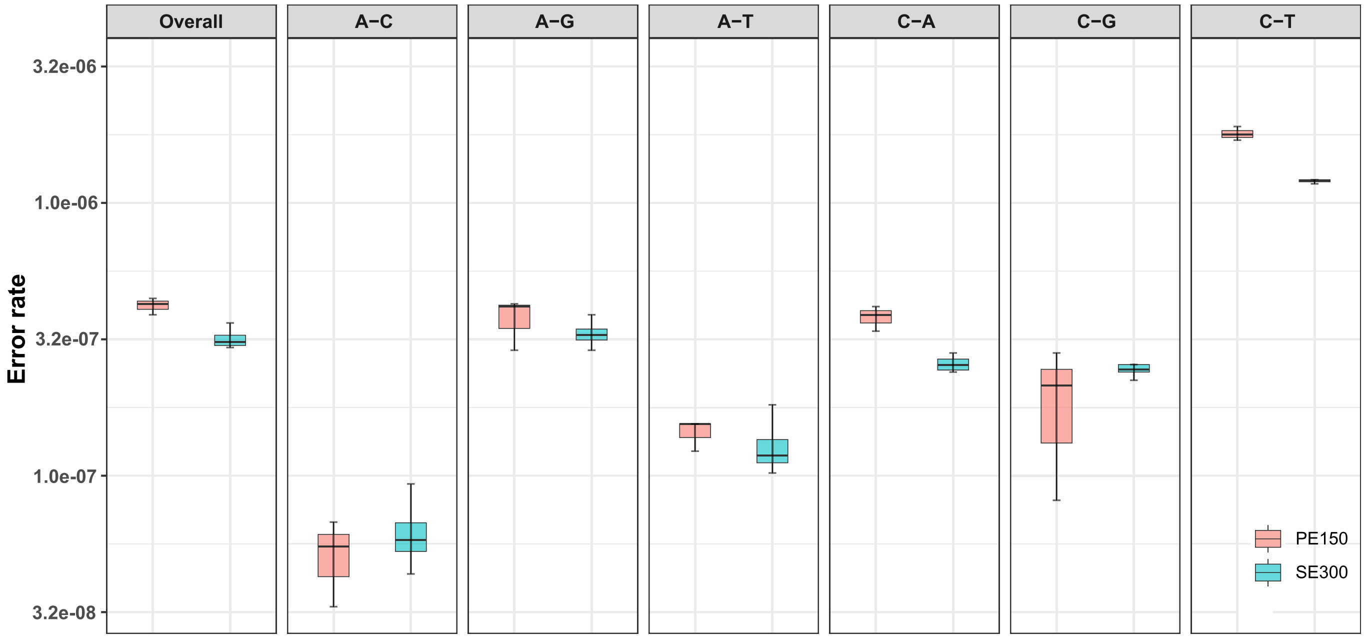

Figure EV1. AccuScan error rate.

Overall error rate and error rate of each variant type from AccuScan whole genome sequencing data on healthy human cell-free DNA samples ($n = 3$) sequenced by pair end 150 (PE150) or single end 300 (SE300) using the 300 cycle sequencing reagents. The line in the middle of the boxplot represents the median value; the box borders reflect the interquartile range (IQR, 25th to 75th percentiles); and the whiskers indicate 1.5 times IQR, with the lower line representing 25th percentile − 1.5*IQR and the upper line representing 75th percentile + 1.5*IQR.

 © The Author(s)

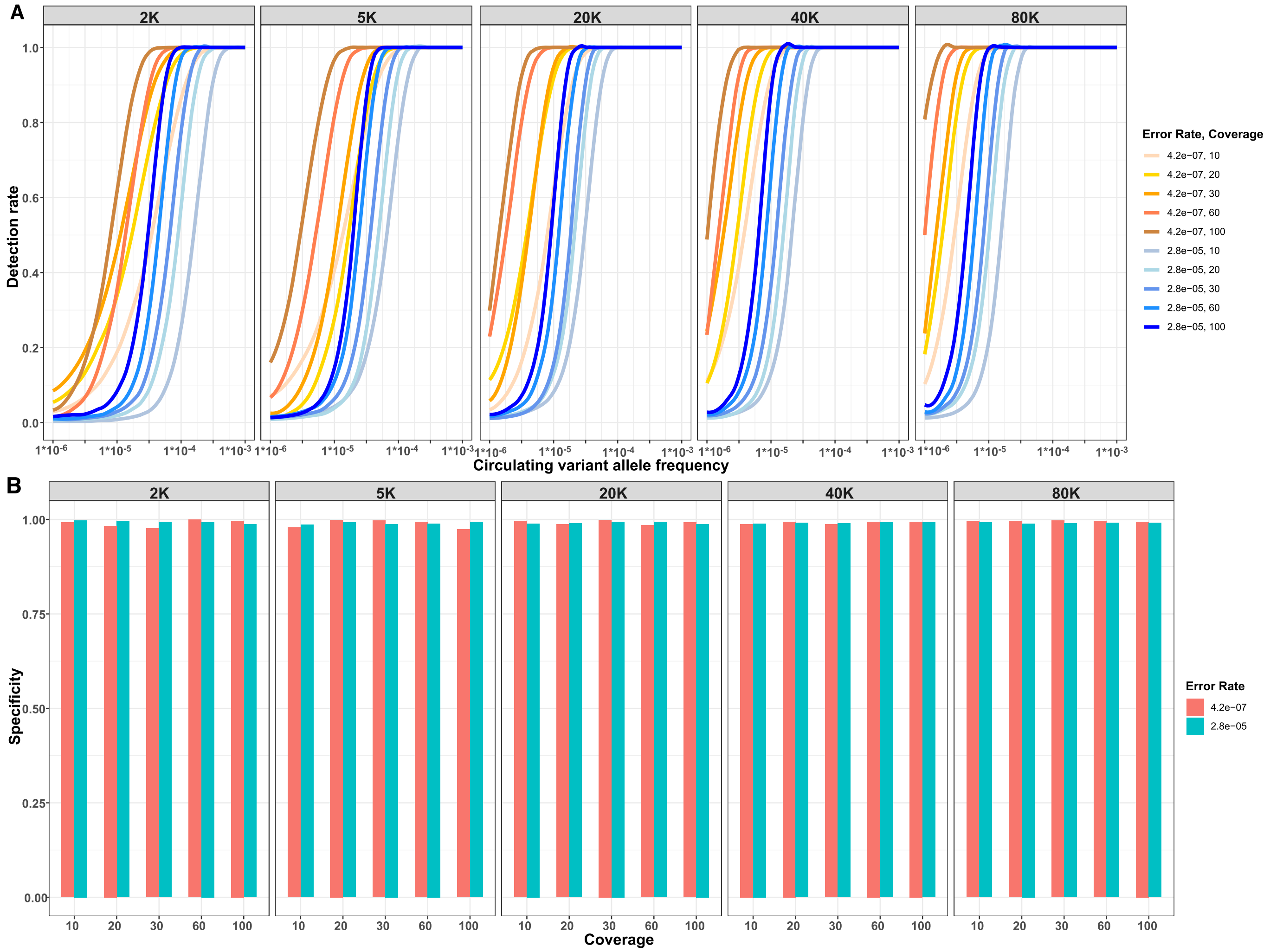

Figure EV2. Analytical sensitivity and specificity of AccuScan.

(**A**) Simulation using 2K, 5K, 20K, 40K, 80K markers and two different error rates to predict the theoretical detection rate under different sequencing coverages as a function of circulating variant allele frequency (cVAF). The 4.2×10^{-7} error rate showed higher sensitivity than the 2.8×10^{-5} error rate under the same sequencing depth. Detection rate is calculated as the fraction of tests that are called molecular residual disease (MRD) positive with the nominal specificity set at 99%. (**B**) Simulation using 2K, 5K, 20K, 40K, 80K markers and two different error rates to predict the theoretical specificity with the nominal specificity setting at 99%. Specificity is calculated as the fraction of tests that are called MRD negative when cVAF is zero.

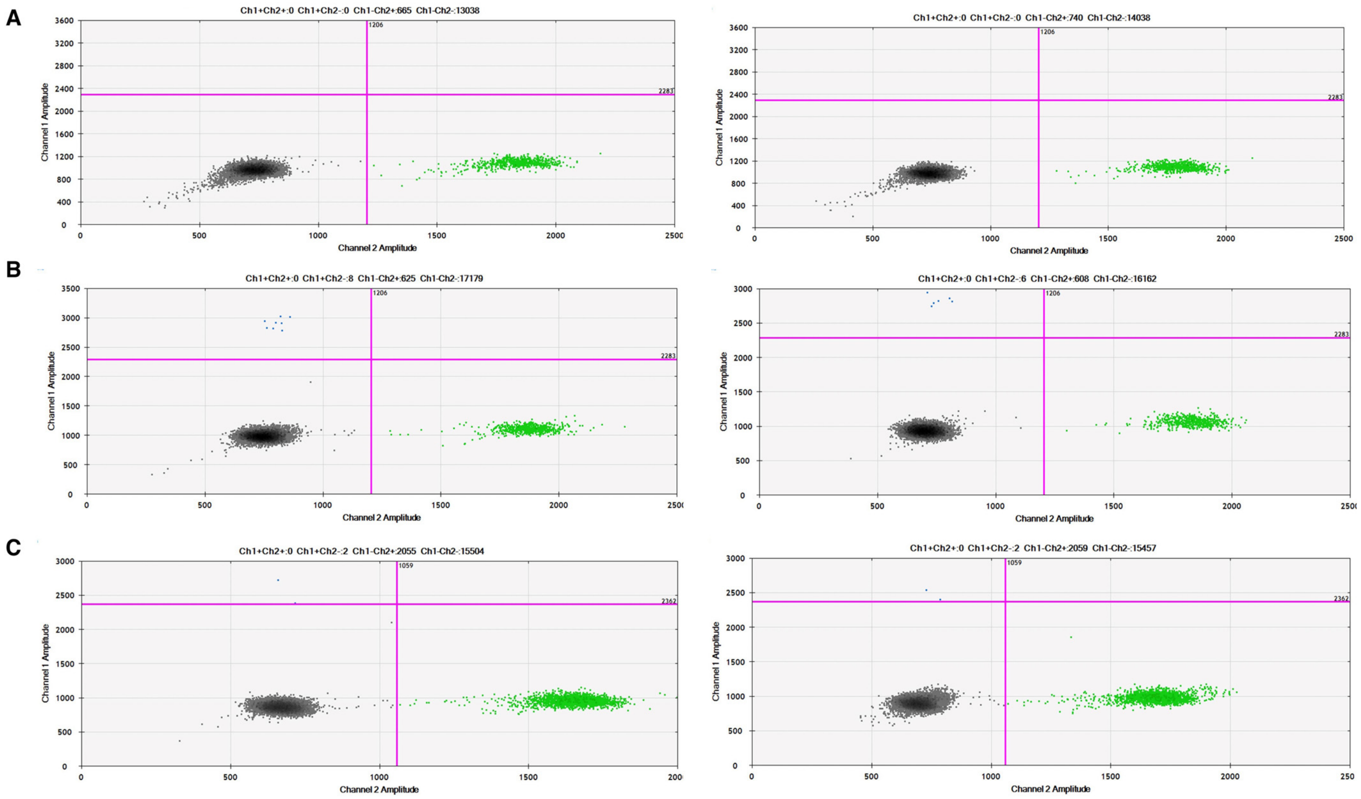

Figure EV3. Droplet digital PCR of the melanoma cancer cell-free DNA sample.

(**A**) Droplet digital PCR (ddPCR) analysis of a healthy plasma sample. (**B**) ddPCR analysis of the original melanoma cancer cell-free DNA (cfDNA) sample using the BRAF V600E assay. (**C**) ddPCR of the diluted melanoma cancer cfDNA sample in the healthy plasma background at an expected circulating variant allele frequency of 0.1%. Each shows the results of two independent replicates of ddPCR tests.

 © The Author(s)

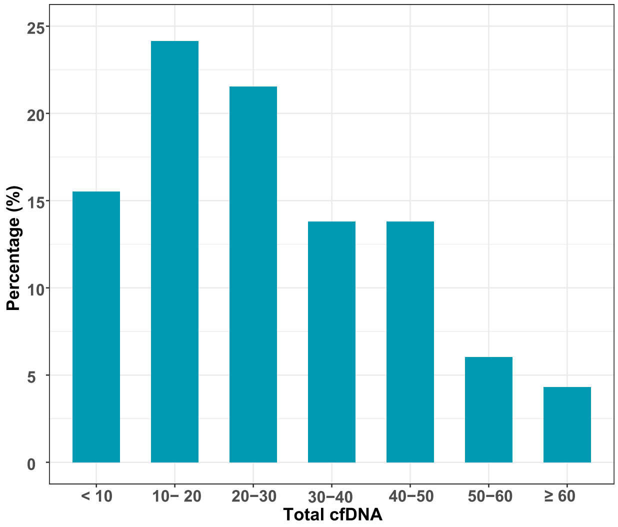

Figure EV4. Histogram of total cell-free DNA yield from plasma samples used in this study.

Cell-free DNA (cfDNA) was extracted from 1 to 4 mL of plasma.

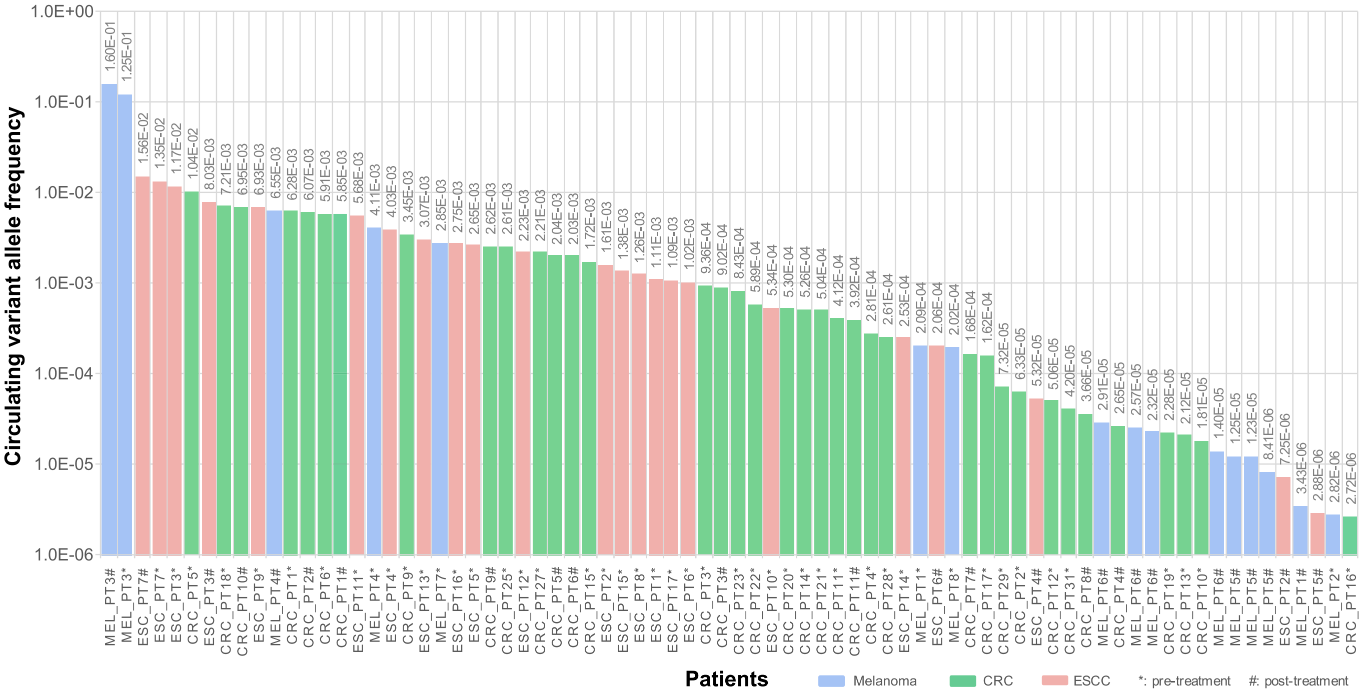

Figure EV5. Circulating variant allele frequency of all circulating tumor DNA positive plasma samples.

Plot included pre-treatment and post-treatment samples. CRC colorectal cancer, ESCC esophageal squamous cell carcinoma.

© The Author(s)
